# Redox homeostasis protects mitochondria through accelerating ROS conversion to enhance hypoxia resistance in cancer cells

**DOI:** 10.1038/srep22831

**Published:** 2016-03-09

**Authors:** Pengying Li, Dongyang Zhang, Lingxiao Shen, Kelei Dong, Meiling Wu, Zhouluo Ou, Dongyun Shi

**Affiliations:** 1 Department of Biochemistry and Molecular Biology, Shanghai Medical College of Fudan University, Shanghai 200032, People’s Republic of China; 2 Cancer Research Institute of Fudan University Shanghai Cancer Center, Shanghai 200032, People’s Republic of China

## Abstract

Mitochondria are the powerhouses of eukaryotic cells and the main source of reactive oxygen species (ROS) in hypoxic cells, participating in regulating redox homeostasis. The mechanism of tumor hypoxia tolerance, especially the role of mitochondria in tumor hypoxia resistance remains largely unknown. This study aimed to explore the role of mitochondria in tumor hypoxia resistance. We observed that glycolysis in hypoxic cancer cells was up-regulated more rapidly, with far lesser attenuation in aerobic oxidation, thus contributing to a more stable ATP/ADP ratio. In hypoxia, cancer cells rapidly convert hypoxia-induced O_2_·^−^ into H_2_O_2_. H_2_O_2_ is further decomposed by a relatively stronger antioxidant system, causing ROS levels to increase lesser compared to normal cells. The moderate ROS leads to an appropriate degree of autophagy, eliminating the damaged mitochondria and offering nutrients to promote mitochondria fusion, thus protects mitochondria and improves hypoxia tolerance in cancer. The functional mitochondria could enable tumor cells to flexibly switch between glycolysis and oxidative phosphorylation to meet the different physiological requirements during the hypoxia/re-oxygenation cycling of tumor growth.

Hypoxia—a reduction in the normal level of tissue oxygen tension—produces cell death if severe or prolonged. It exists in some parts of solid tumors because of incomplete blood vessel networks and the imbalance between proliferation and angiogenesis^1^,^2^. A mounting body of evidences demonstrated that a hypoxic microenvironment is coincident with the development and maintenance of tumors^3^. Although hypoxia is toxic to both cancer cells and normal cells, cancer cells survive, proliferate and gain resistance to radiation and chemotherapy in a hypoxic environment, by undergoing genetic and adaptive changes^4^. These processes contributed to the malignant phenotype and aggressive tumor behavior, causing poor prognosis^5^,^6^.

Cellular responses to hypoxia include processes that enhanced oxygen delivery, increased glucose transport, increased glycolytic metabolism, and switching oxidative phosphorylation to anaerobic glycolysis^7^. Therefore, cancer cells undergo an aberrant metabolic shift to glycolytic energy dependence in the presence of oxygen – the so-called “Warburg effect” or “aerobic glycolysis”^8^. Initial studies suggested that respiratory impairment or suppression leads to Warburg effect^9^. However, recent research shows that cancer cells exhibited protection from apoptosis under hypoxia and is associated with enlarged but functional mitochondria^10^, which raise the question as to whether mitochondria lost all their functions. To date, how mitochondria in cancer cells respond to hypoxia, and whether there is a difference between normal cells and cancer cells, remain elusive.

Hypoxic cells are threatened by excessive ROS accumulation and decreased mitochondrial ATP production, which could be alleviated by Warburg effect^11^. Hypoxia tolerance is a process that cancer cells use to adapt to both energy depletion process and ROS attacks. Mitochondria, the powerhouse of eukaryotic cells and the main source of ROS in hypoxic cells, participate in redox homeostasis regulation^12^. Since most tumor cells are resistant to hypoxia induced apoptosis, we speculated that a mechanism in hypoxic cancer cells, mediated by mitochondria, might exist to regulate metabolism and redox homeostasis, making cancer more tolerant to hypoxic microenvironment.

In this study we mimic the tumor hypoxic microenvironment *in vitro* by culturing cells in a tri-gas incubator with an oxygen concentration of 0.2%. By comparing the responses to hypoxia between normal cell lines and cancer cell lines, we attempt to identify possible ways that only exist in cancer cells when coping with hypoxia stress, reveal important roles of redox homeostasis and mitochondria in elevating hypoxic tumor survival rates, and offer a new explanation of tumor hypoxia tolerance.

## Results

### Cancer cells have higher survival rates under hypoxia

**Figure 1:**
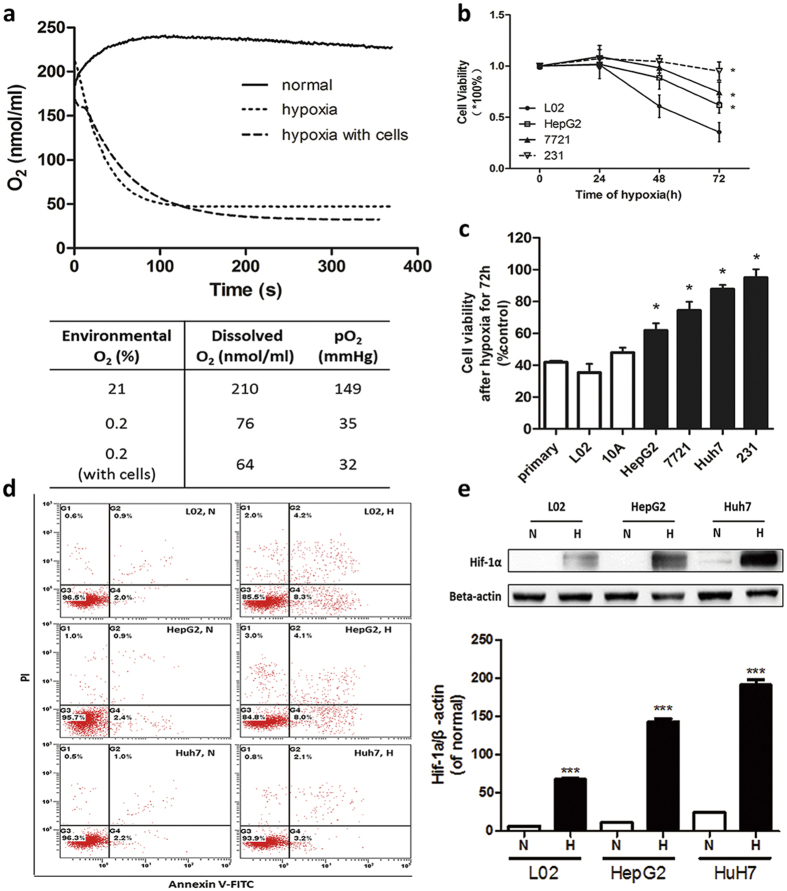
Cancer cell lines have higher rates of hypoxia survival than normal cell lines. (**a**) Dissolved oxygen concentration of DMEM equilibrated in 21% O_2_ incubator (solid line) or 0.2% O_2_ incubator (open line) for 24 hours. The corresponding data was exhibited in the chart below. (**b**) Time-dependent inhibition of different cell lines’ viability using MTT assay. (**c**) The viability of different cell lines in 0.2% O_2_ for 72 h using MTT assay. (**d**) Analysis of cell apoptosis of L02, HepG2 and Huh7 under hypoxia by annexin V/PI double-staining assay. Representative FACS analysis scattergrams of annexin V–FITC/PI staining are shown on the left, the statistical data on the right. N, 20% O_2_ for 24 h; H-24 h, 0.2% O_2_ for 24 h. Results are shown as mean ± SD, n = 3, *p < 0.05 *versus* L02. (**e**) Representative western blot and the quantification analysis of HIF-1a. N, normoxia; H, hypoxia for 24 h. Results are shown as mean ± SD, ***p < 0.001 *versus* the normal group of each cells, n = 3.

To date, most studies on cancer *in vitro* are conducted in regular incubators with 20% oxygen concentration (oxygen partial pressure: 149 mmHg), which is higher than the physiological value of normal tissue - 60 mmHg, and is much higher than the depressed 15 mmHg in hypoxic cancer tissue^13^,^14^. In order to genuinely reflect the microenvironment in solid tumors, we utilized a tri-gas incubator with oxygen concentration in 0.2%, oxygen partial pressure: 32 mmHg (Fig. 1a). This hypoxic culture condition mimics tumor hypoxic condition during carcinogenesis.

Various cell lines are used to investigate hypoxia effect on cell viability including non-cancerous cells (primary hepatocytes, L02, MCF-10A), less malignant cancer cells (p53 wild type HepG2 and SMMC-7721), highly malignant cancer cells (p53 mutant Huh7 and MDA-MB-231). It is know that p53 genetic mutation was associated with more aggressive tumors^15^,^16^,^17^,^18^. Notably, Huh7 and MDA-MB-231 (cell lines carried mtp53) have the highest survival rates under hypoxia, HepG2 and SMMC-7721(cancer cell lines with wtp53) take second place, while L02 and MCF-10A (normal cell lines) are significantly restrained (Fig. 1b,c). Similarly, the apoptosis rates of hypoxic Huh7 are lower than L02 and HepG2 (Fig. 1d). Western blot shows that Hif1-alpha in Huh7 was upregulated much more significantly in response to hypoxia (Fig. 1e). These results suggest that p53 mutation may confer cancer cells more tolerance to hypoxia, which is consistent with a previous study^19^.

### More rapidly up-regulated glycolysis and less attenuation in aerobic oxidation contribute to a more stable ATP/ADP ratio in hypoxic cancer cells

**Figure 2:**
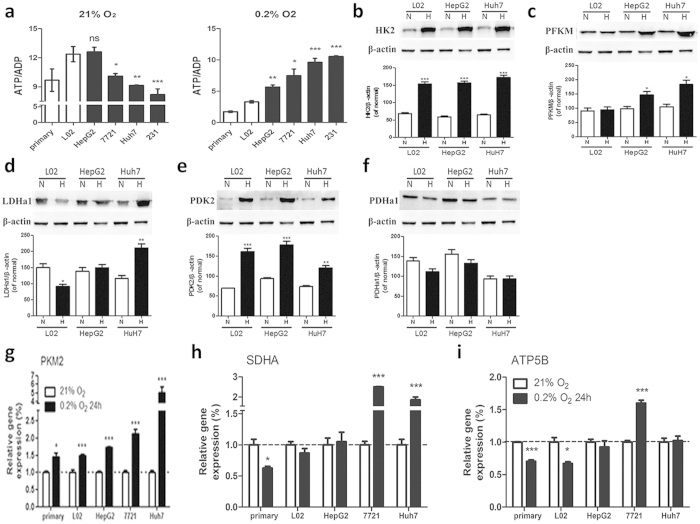
More rapidly up-regulated glycolysis and less attenuation in aerobic oxidation contribute to a more stable ATP/ADP ratio in hypoxic cancer cells. (**a**) ATP/ADP levels of different cell lines. NS, no significance; *p < 0.05; **p < 0.01; ***p < 0.005 *versus* L02. n ≥ 3. (**b–f**) Representative western blot and the quantification analysis of glycolysis-related enzymes. N, normoxia; H, hypoxia for 24 h. *p < 0.05; **p < 0.01; ***p < 0.005 *versus* the normal group of each cells, n = 3. RT-PCR analysis of relative. (**g**) PKM2, (**h**) SDHA and (i)ATP5B mRNA levels normalized to HPRT mRNA levels. Results are expressed as fold changes from control. White column, normoxia; Black column, hypoxia for 24 h. Results shown as mean ± SD, n ≥ 3, *p < 0.05, **p < 0.01, ***p < 0.005 *versus* control.

We suspect that cancer cells may have the ability to produce enough ATP even under hypoxia condition. ATP/ADP ratio presents the ability of ATP synthesis. High malignant cancer cells, Huh7 and 231 possess the highest ATP/ADP ratio in hypoxic state, followed by less malignant cancer cells HepG2, and finally, non-cancerous cells, the primary hepatocytes and L02 (Supplementary infomation Table S2). Interestingly, ATP production in non-cancerous cells/less malignant cells is higher than the more malignant ones when the oxygen is sufficient (Fig. 2a left), highly malignant cells, nonetheless, have the highest ATP level (Fig. 2a right) under hypoxia conditions.

After analyzing glycolysis enzymes: phosphofrutokinase-1(PFK-1, also known as PFKM), pyruvate kinase muscle isozyme (PKM), lactate dehydrogenase A (LDHA), and hexokinase 2 (HK2) in response to hypoxia, results showed that glycolysis was significantly upregulated in cancer cells, especially in the highly malignant cells (Fig. 2b–d,g). This indicates that rapidly up-regulated glycolysis may contribute to relatively stable ATP production in hypoxic cancer cells. We further examined alterations to aerobic oxidation pathways in response to hypoxia. Our results showed that pyruvate dehydrogenase kinase (PDK), a pyruvate dehydrogenase (PDH) inactivator, increased under hypoxia, however the highly malignant cells (Huh7) increased much less than non-cancerous/less malignant cells, meanwhile the PDH expression in cancer cells was well maintained, ATP5B (ATP synthase subunit beta) and succinic dehydrogenase (SDH) was significantly upregulated in cancer cells but they all slightly decreased in non-cancerous cells (Fig. 2e,f,h,i). These results indicated that cancer cells conserve aerobic oxidation function.

### The enhanced tolerance to hypoxia is associated with the maintenance of mitochondria ultrastructure and function

**Figure 3:**
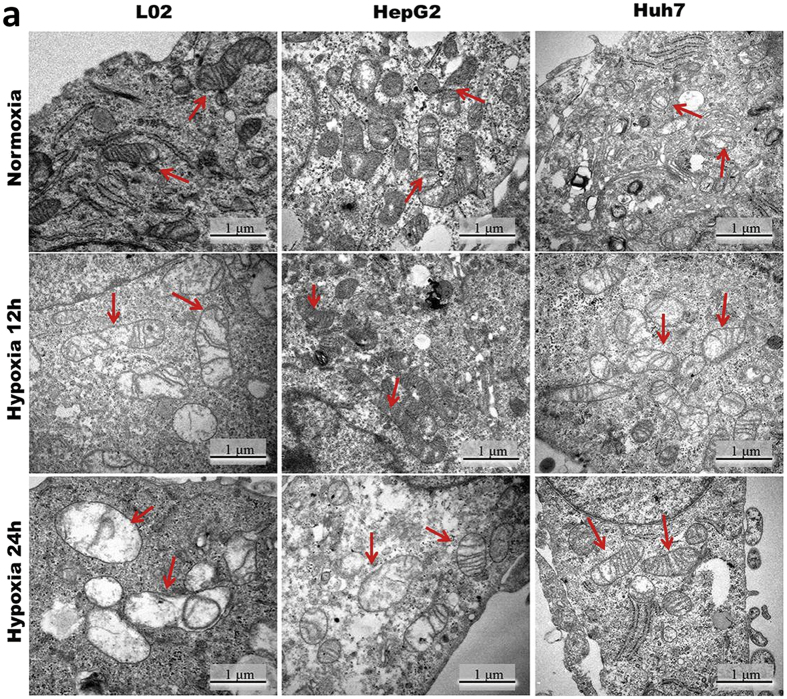
The enhanced tolerance to hypoxia is associated with the maintenance of mitochondria ultrastructure. (**a**) Representative electron micrographs of L02 HepG2 and Huh7 cells (Normoxia 21% O_2_ (top part); Hypoixa 0.2% O_2_ for 12 h (middle part) and 24 h (bottom part)), n = 3. Arrows denote healthy or impaired mitochondria.

Well-functioning aerobic oxidation pathways and ATP5B in hypoxic cancer cells shifted our focus to the mitochondria state in oxygen deprivation. Mitochondria ultrastructure was studied by transmission electron microscopy (TEM). In a normoxic state, mitochondria in non-cancerous L02 cells are well-arranged and distributed evenly and double membrane structure is clear, without obvious swelling, while mitochondria in cancer cells (HepG2 and Huh7) are slightly swollen with a relatively blurry double membrane structure. After 12 hours hypoxia, the mitochondria of L02 showed swelling, crista fragmentation and degeneration. On the contrary, although some autophagy vacuoles existed in cancer cells, most mitochondria were in better condition under hypoxia (≥24 h), especially in the highly malignant cells (Fig. 3a).

**Figure 4:**
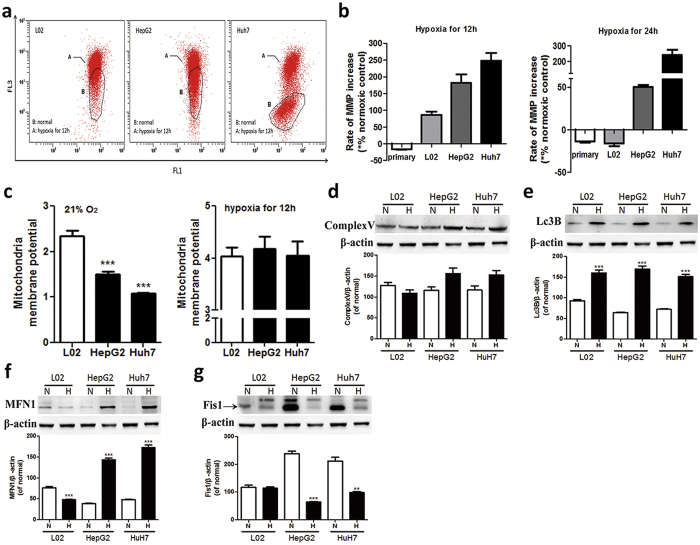
The enhanced tolerance to hypoxia is associated with the maintenance of mitochondria function. (**a**) Flow cytofluorimetry of L02, HepG2 and Huh7 cells, in normoxia (21% O_2_, 12 h, region B), and hypoxia (0.2% O_2_, 12 h, red scatter plot), stained with JC-1. The corresponding data was exhibited in (**b**), n ≥ 3. (**c**) Rate of mitochondria membrane potential increase after hypoxia for 12 h (left part) and 24 h (right part) by JC-1 assay, n ≥ 3. (**d–g**) Representative western blot and the quantification analysis of mitochondrial function related protein. N, normoxia; H, hypoxia for 24 h. Results are shown as mean ± SD, **p < 0.01, ***p < 0.001 *versus* the normal group of each cells, n = 3.

Mitochondrial membrane potential (MMP, Ψm) is the main indicator of mitochondrial metabolism^20^. Cancer cells’ MMP is lower than non-cancerous cells, indicating that their mitochondria are abnormal in normoxic state in normoxic state (Fig. 4a,b). Interestingly, L02 cells undergo a small spike in MMP at around 12 h for hypoxia then rapidly drop below its normoxic value as primary hepatocytes, whereas MMP level in HepG2 and Huh7 cells elevate rapidly and maintain it for at least 24 h under hypoxia (Fig. 4c).

We further analyzed mitochondria-related proteins in response to hypoxia, including the Complex V, mitochondrial fusion related protein (MFN), mitochondrial fission-related protein (Fis), mitochondrial autophagy protein (LC3B) (Fig. 4d–g). Our results showed that LC3B increased in all cells, which is consistent with the visible autophagy vacuoles in TEM images. Despite that, cancer cells showed an increment in mitochondrial fusion but decrement in mitochondrial fission, together with Complex V increase. These results support our speculation that mitochondrial function in cancer cells is well maintained under hypoxia.

### Cancer cells undergo much smaller growth in ROS level after hypoxic stress

**Figure 5:**
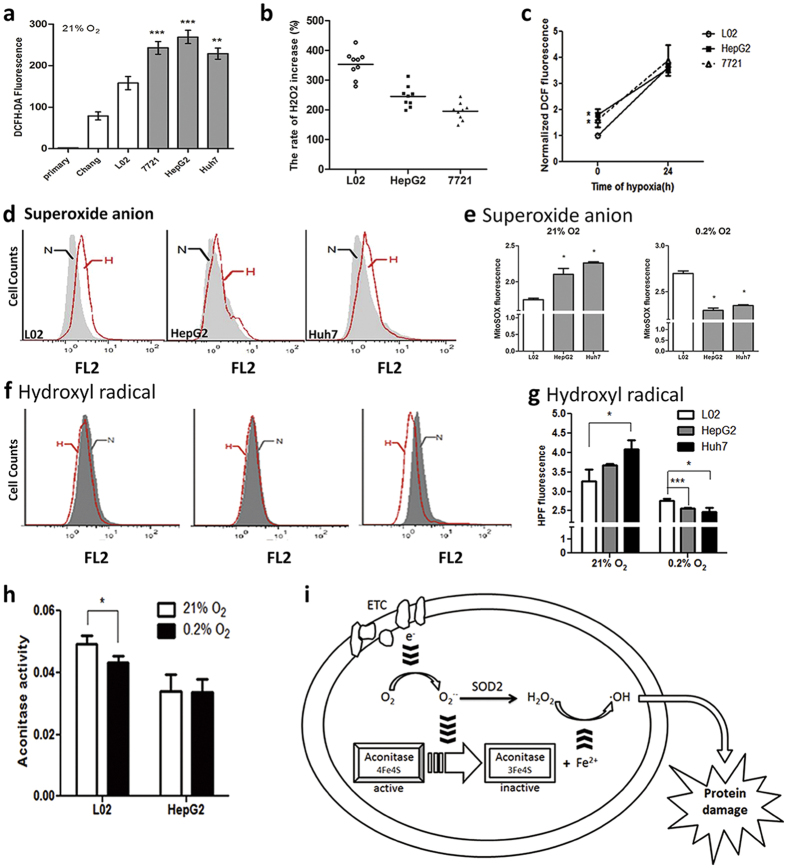
Cancer cells undergo a much smaller growth in ROS level after hypoxic stress. (**a**) Intracellular H_2_O_2_ production in 21% O_2_ determined by FCM with DCFH-DA (λex = 488 nm, λem = 525 nm). Results are shown as mean ± SD, **p < 0.01 *versus* L02. n ≥ 3. (**b**) Rate of H_2_O_2_ increase after hypoxia for 24 h by FCM with DCFH-DA. Results are shown as mean ± SD, **p < 0.01 *versus* L02. n ≥ 3. (**c**) Normalized intracellular H_2_O_2_ production after hypoxia for 24 h by FCM with DCFH-DA. Results are shown as mean ± SD, *p < 0.05 *versus* L02. n ≥ 3. (**d**) and (**e**) Intracellular superoxide anion production under normoxia or after hypoxia for 24 h measured by FCM with the MitoSOX. Results are shown as mean ± SD, *p < 0.05 *versus* L02. n ≥ 3. (**f**) and (**g**) Intracellular hydroxyl radical production of L02, HepG2 and Huh7 after normoxia or hypoxia for 24 h measured by FCM with HPF. Results are shown as mean ± SD, *p < 0.05, ***p < 0.001 *versus* L02. n ≥ 3. (**h**) Aconitase activity of L02, HepG2 and Huh7 after incubation under normoxia or hypoxia for 24 h. White column, normoxia; Black column, hypoxia for 24 h. Results are shown as mean ± SD, *p < 0.05, *versus* L02. n ≥ 3. (**i**) Under hypoxia, O_2_ •− oxidizes aconitase and promotes formation of hydroxyl radicals, thus damages the mitochondria components and cellular protein.

Although cancer cells initially display higher intracellular H_2_O_2_ levels as indicated by DCF fluorescence than non-cancerous cells (primary hepatocytes, Chang, L02) in normoxic state (Fig. 5a), the increase is very weak after 24 hours hypoxia. H_2_O_2_ levels in L02 rises sharply in hypoxic environment (Fig. 5c), and the increase rate is nearly twice in L02 than cancer cells (Fig. 5b). Interestingly, the O_2_·^−^ and ·OH accumulation in cancer cells is significantly higher than L02 in normoxic state but significantly lower than L02 in hypoxic state (Fig. 5d–g). Meanwhile mitochondrial aconitase activity in L02 cells was inhibited but it was well-maintained in cancer cells (Fig. 5h). Aconitase is highly sensitive to ROS which inactivates the enzyme to release Fe^2+^ 21, Fe^2+^ could further convert H_2_O_2_ to more toxic ·OH, thus damage protein^22^. We speculate that lower O_2_·^−^ and ·OH level in hypoxic cancer cells could prevent mitochondria-related protein from ROS damage thus contributing to the better mitochondria (Fig. 5i).

### Antioxidant signal, enzymes and intracellular redox buffering are enhanced in hypoxic cancer cells

**Figure 6:**
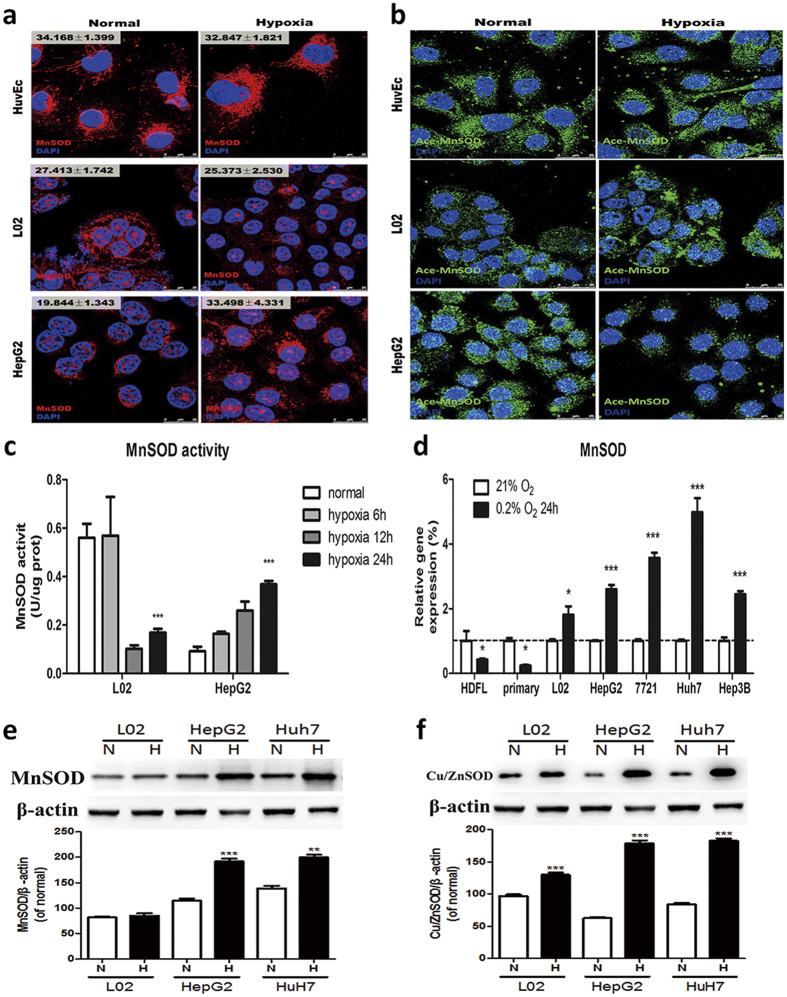
MnSOD helped to maintain redox homeostasis in hypoxic cancer cells. Representative images of immunofluorescence detection labeled with the (**a**) anti-MnSOD antibody and (**b**) anti-acetylated MnSOD antibody. Nuclei were stained with DAPI. Scale bar, 25 μm. Related intensities are shown as mean ± SD, n ≥ 3. (**c**) Activity of MnSOD in L02 and HepG2 cells determined by a WST-1 assay. Cells were incubated in 0.2% O_2_ for 6 h, 12 h or 24 h. Results are shown as mean ± SD, ***p < 0.001 *versus* L02. n ≥ 3. (**d**) RT-PCR analysis of MnSOD in different cell lines by comparing to their normoxia control. *p < 0.05, ***p < 0.001 *versus* the normal group of each cells, n ≥ 3. (**e**) and (**f**) Representative western blot and the quantification analysis of MnSOD and Cu/ZnSOD. N, normoxia; H, hypoxia for 24 h. Results are shown as mean ± SD, **p < 0.01, ***p < 0.001 *versus* the normal group of each cells, n = 3.

**Figure 7:**
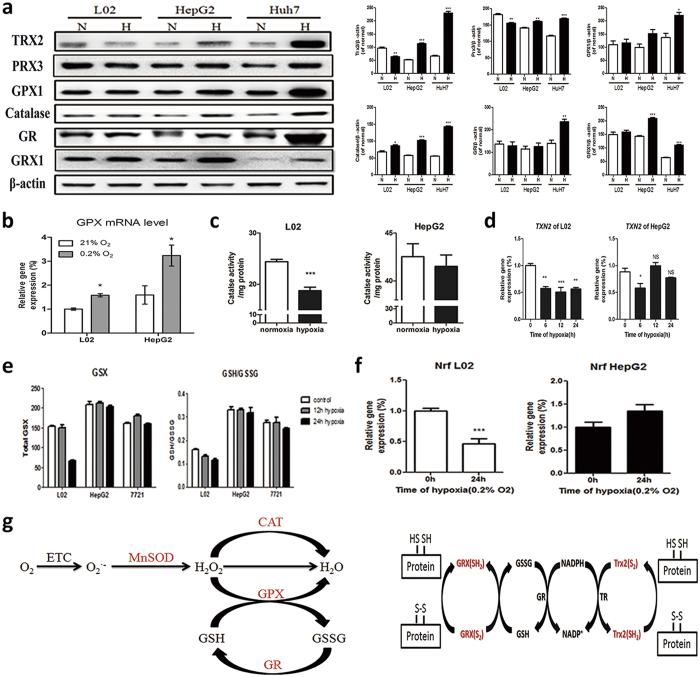
Antioxidant signal, enzymes and intracellular redox buffering are enhanced in hypoxic HepG2. (**a**) Representative western blot analysis of the antioxidant proteins and enzymes. L02, HepG2 and Huh7 cultured in 21% or 0.2% O_2_ for 24 h. Results are shown as mean ± SD, *p < 0.05, **p < 0.01, ***p < 0.001 *versus* the normal group of each cells, n = 3. (**b**) RT-PCR analysis of GPX in L02 and HepG2 cultured under normoxia or after hypoxia for 24 h. Results are shown as mean ± SD, **p < 0.01 *versus* the normal group, n ≥ 3. (**c**) Catalase activity of L02 and HepG2 cells cultured in normoxia or hypoxia for 24 h. Results are shown as mean ± SD, ***p < 0.001 *versus* the normal group, n ≥ 3. (**d**) RT-PCR analysis of Trx2 in L02 and HepG2 cultured after hypoxia for 0 h, 6 h, 12 h and 24 h. Results are shown as mean ± SD. NS, no significance, *p < 0.05, **p < 0.01, ***p < 0.001 *versus* 0 h group, n ≥ 3. (**e**) Total GSX and GSH/GSSG ratios in L02, HepG2 and 7721 which was assayed using OPA fluorescence. Results are shown as mean ± SD, n ≥ 3. (**f**) The relative mRNA expression of Nrf2 in L02 and HepG2 after hypoxia for 0 h and 24 h. Results are shown as mean ± SD. ***p < 0.001 *versus* normal, n ≥ 3. (**g**) Cancer cells’ antioxidant system was upregulated in response to hypoxia.

Within the mitochondrial matrix, MnSOD is an essential antioxidant enzyme that catalyzes the conversion of O_2_·^−^ to H_2_O_2_^23^. Inhibition of SOD causes cellular O_2_·^−^ accumulation, causing mitochondria damage and cell apoptosis^24^. MnSOD expression (Fig. 6a,d,e) and activity (acetylation at Lys68 decreases MnSOD activity)^25^ (Fig. 6b,c) all raised in paralleled with increased Cu/ZnSOD expression in cancer cells (Fig. 6f), but decreased in non-cancerous cells (L02, HuvEc, HDF, primary hepatocytes) after hypoxia. We further measured the other cellular redox proteins in response to hypoxia (Fig. 7g). As we expected, antioxidant systems including Catalase (CAT), peroxidase (Prx), glutathione peroxidase (Gpx), glutathione reductase (GR), glutaredoxins (Grxs), thioredoxins (Trxs), NF-E2-related factor 2 (Nrf2) were all upregulated in cancer cells in response to hypoxia, but they decreased or are unchanged in non-cancerous cells (Fig. 7a–d,f). Highly malignant cells presented higher antioxidant protein level compared to less malignant cells. All these suggest that cancer cell have a powerful antioxidant response under hypoxia.

GSH/GSSG is an important cellular redox buffering system. Both GSH/GSSG ratio and GSX (GSH + GSSG) were significantly higher than that in non-cancerous cells under hypoxia (Fig. 7e). We further analyzed the expression of glutamate-cysteine ligase (GCLC), glutathione synthetase (GSS) and glutaminase 2 (GLS2). All are involved in catalyzing the formation of GSH or GSH’s precursors. GLS2, which catalyzes glutamine to glutamate, was significantly increased in hypoxic HepG2 cancer cells (Supplementary Fig. S1). This may contribute to the total (GSH + GSSG) level increase in cancer cell under hypoxia, which confers cancer cells a higher redox capacity.

### G6PD and IDH2 helped to maintain redox homeostasis in hypoxic cancer cells

**Figure 8:**
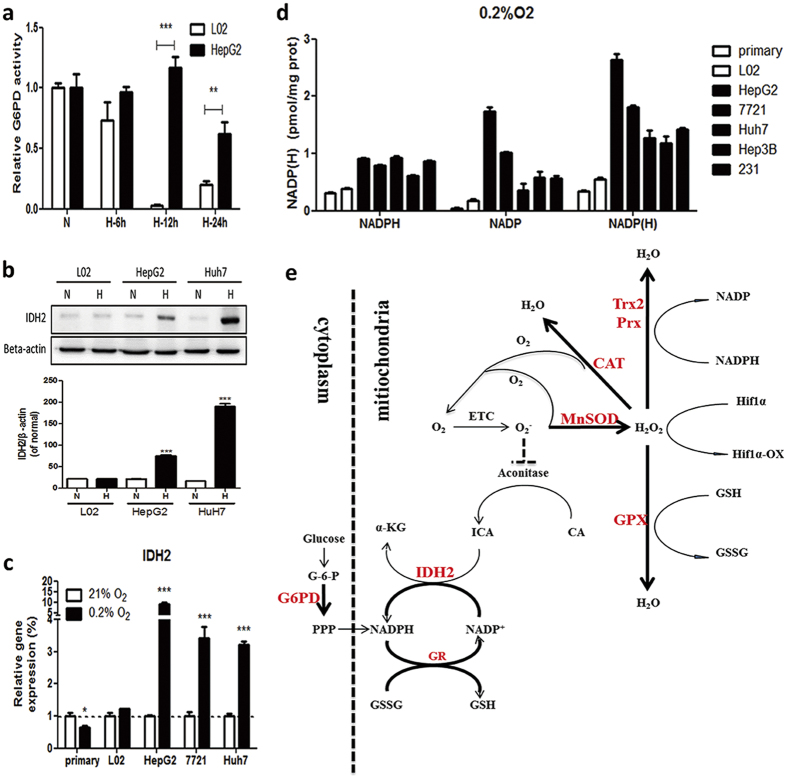
IDH2 and G6PD were involved in cancer hypoxia tolerance. (**a**) G6PD activity of L02 and HepG2 under normoxia or hypoxia. N, normoxia; H-6 h, hypoxia for 6 h; H-12 h, hypixia for 12 h; H-24 h, hypoxia for 24 h. Results are shown as mean ± SD. **p < 0.01, ***p < 0.001 *versus* L02, n ≥ 3. (**b**) Representative western blot and the quantification analysis of IDH2. N, normoxia; H, hypoxia for 24 h. Results are shown as mean ± SD, ***p < 0.001 *versus* the normal group of each cells, n = 3. (**c**)The relative mRNA level of IDH2 in L02 and HepG2 cells after being cultured under normoxia (21% O_2_) or hypoxia (0.2% O_2_). Results are shown as mean ± SD. *p < 0.05, ***p < 0.001 *versus* normal, n ≥ 3. (**d**) NADPH level in different cell lines cultured in 0.2% O_2_ for 24 h. Normal cell line, white bar; cancer cell line, black bar. Results are shown as mean ± SD, n ≥ 3. (**e**) Cancer cells may themselves reduce redundant H_2_O_2_ and provide more NADPH and TCA cycle intermediates to maintain hypoxia survivability through G6PD and IDH2 pathways.

Glutathione system needs NADPH to maintain its reduced state, which plays an important role in the maintenance of redox homeostasis in cells^26^. As shown in Fig. 8d, NADPH also remains higher in cancer cells than non-cancerous cells under hypoxia, which contributes to the higher GSH/GSSG ratio in hypoxic HepG2 cells. The major source of NADPH is the pentose phosphate pathway, in which G6PD acts as a key enzyme. G6PD activity drops rapidly in L02 after 12 h hypoxia but remains relative stable in HepG2 (Fig. 8a). Isocitrate dehydrogenase 2 (IDH2), a NADP^+^-dependent isocitrate dehydrogenase localize to the mitochondria^27^, exhibits unusual increases after hypoxia in cancer cells (Fig. 8b,c). Given that IDH2 also performs catalytic actions on isocitric acid, by dehydrogenating it to form α-ketoglutarate and NADPH, we speculate that cancer cells may themselves provide more NADPH and TCA cycle intermediates to maintain hypoxia survivability through G6PD and IDH2 pathways (Fig. 8e).

## Discussion

In this study, we explored the mechanism of hypoxia tolerance using various cell lines (including primary mice liver cells, normal and cancer cell lines from human liver, lung, breast and stomach, with wild or mutant p53). We demonstrate that under hypoxia, ATP/ADP ratio in cancer cell lines are generally higher than that in normal cell lines; it is much higher in highly malignant tumor cells with mutant p53, which is consistent with their hypoxia tolerance ability. The relative stable ATP/ADP ratio provides more energy to hypoxic cancer cells, which may be a significant reason why cancer cells have survival advantages under hypoxia. Our previous results have shown that cancer cells possess high level of glycolysis after experiencing varying degrees of hypoxia^28^. However, respiration dysfunction will lead to more electrons leak and superoxide anion production, even when oxygen is sufficient. This further impairs cell components. This raises the question regarding how tumor cells survive relying on low efficiency glycolysis and dealing with hypoxia-induced oxidative stress.

We found that, although the aerobic metabolic capacity in cancer cells also decreased, it was nevertheless maintained at a relatively stable and higher level than that of normal cells. Due to the oxygen deprivation, mitochondrial aconitase, SDH, IDH2 and ATP5B dropped significantly in non-cancerous cells but remained stable or even increased in hypoxic cancer cells. As the primary regulators of aerobic oxidation, PDH expression was well-maintained in cancer cells under hypoxia. PKD2 inactivates PDH and this plays a central role in regulating glycolysis and oxidative phosphorylation^29^. Our results showed that PDK2 expression increase was much lesser in cancer cells under hypoxia, herein indicating the greater PDH activity in cancer cells than that in non-cancerous cells. This suggests that hypoxic cancer cells conserve the aerobic oxidation function. Thus, rapidly up-regulated glycolysis with functional aerobic oxidation collaboratively contributes to a more stable ATP/ADP ratio, and avoids energy failure in cancer cells.

Our results show that the cancer cells’ mitochondria are abnormal in normoxic state. Interestingly, in a hypoxic state, cancer cells’ mitochondria do not deteriorate further, however, it is seriously impaired in non-cancerous cells. By comparing different kinds of tumor cell lines, we noticed that the highly malignant cancer cell lines, whose mitochondria is less sensitive to hypoxic stress and maintained its functionality, even when experiencing a prolonged hypoxia. We also examined the mitochondrial related proteins. Mfn1, a mitochondrial protein involved in mitochondrial fusion^30^, increased in cancer cells in hypoxia compared to normoxia but decreased in non-cancerous cells. Conversely, Fis1, implicated in the regulation of mitochondrial fission^31^, decreased in cancer cells. Increased fusion and decreased fission are implicated in better mitochondrial function^32^. These results were consistent with the phenomena that cancer cells’ mitochondria were enlarged in TEM image under hypoxia. These results strongly supported that cancer cells’ mitochondria still have function. This suggests the important role mitochondria may play in cancer hypoxia tolerance.

Our previous research has showed that ROS could activate Akt^33^ and up-regulate glycolysis by stabilizing Hif1α, and improve a tumor’s hypoxia tolerance^28^. ROS also plays an important role in mitophagy^34^. We speculated that ROS is involved in cancer hypoxia tolerance. We found that the increase of ROS in cancer cells was significantly less than that in non-cancerous cells under hypoxia, which could be because that the basal level of oxidative stress in cancer cells is much higher than in normal cells. We use a mitochondria-targeted antioxidant MitoPBN^35^ and NOX/flavoprotein inhibitor DPI to suppress mitochondria derived and NADPH oxidase derived ROS respectively. These inhibitors significantly suppress ROS accumulation in cancer cells, and inhibit MMP, suggesting that ROS from different sources participate in cancer hypoxia tolerance (Supplementary Fig. S2a,b). Interestingly, although mitochondrial autophagy protein increased in both cancerous and non-cancerous cells, only non-cancerous cells suffered from a large quantity of autophagy as shown in TEM image, which ultimately led to cell death; cancerous cells presented insignificant amount of autophagy, which could protect cancer cell from hypoxia stress as indicated in literatures^36^. We speculate that it was the different redox environment in cancer and normal cells resulting in their different fate in response to hypoxia. We also found that antioxidant intervention has a different effect on normal cells and tumor cells, which coincided with our previous study^37^. Normal cells hypoxia survivability was improved, but that of tumor cells was inhibited after NAC/alpha-LA intervention, again highlighting different redox microenvironment in tumor and normal cells (Supplementary Fig. S3a,b).

Mitochondria ROS exist in many forms. Mitochondrial electron leak is an important source of endogenous ROS under insufficient O_2_. Super oxide anion (O_2_·^−^) is formed by one-electron reduction of oxygen, which is the initial state of ROS and is not very reactive^38^. O_2_·^−^ is rapidly converted to H_2_O_2_ by MnSOD^39^,^40^. The excessive O_2_·^−^ inactivates aconitase and causes Fe^2+^ release^22^. H_2_O_2_ further reacts with Fe^2+^ and converts to · OH, which is the most active ROS that damages mitochondrial protein, initiates lipid peroxidation and destroys mitochondria^35^ (Fig. 5i). Our data show that the MnSOD expression and aconitase activity is higher and · OH level is lower in cancer cells than that in normal cells, indicating that · OH is not accumulated in the mitochondria of hypoxic tumor cells.

Redox signaling plays an important role in regulating cellular metabolism^41^, our recent study has demonstrated that cancer cells have a higher redox threshold than normal cells, which confers them a higher tolerance and a higher demand to ROS^37^. As the “master regulator” of the antioxidant response^42^, Nrf2 expression in hypoxic HepG2 cells is rapidly up-regulated, indicating that cancer cells possess a more sensitive antioxidant response. Moreover, redox proteins including GPxs, Prxs and CAT, which are responsible for H_2_O_2_ conversion, are all up-regulated in cancerous cells. Such acceleration of H_2_O_2_ decomposition reduces the H_2_O_2_ accumulation for the further Fenton reaction, while up-regulated MnSOD activity rapidly removes O_2_·^−^, avoids aconitase inactivation and decreases Fe^2+^ release. Thus, the level of Fenton reaction is largely attenuated and less ·OH is produced to attack mitochondrial components in cancerous cells.

The glutathione system and thioredoxin/glutaredoxins system constitute the intracellular redox buffering. GSX (GSSG + GSH) increase in cancer cells under hypoxia indicates an enhanced *de novo* glutathione synthesis. Our results show that GLS2, which catalyzes glutamine to glutamate (a glutathione synthesis precursor), is significantly increased in hypoxic cancer cells. Glutamine was abundant in cancer and it supplies cancer with energy^43^,^44^. We speculate that the abundant glutamine in cancer and the increased GLS2 in response hypoxia may contribute to the enhanced *de novo* glutathione synthesis. Thus hypoxia could trigger GSH + GSSG redox buffering capacity increase in cancer cells. Similarly, Trx2/Grx/GR up-regulation also increased cancer cells’ mitochondria redox buffering capacity. Furthermore, a high GSH/GSSG ratio suggests enhanced reducing capacity in hypoxic cancer cells, emphasizing lower oxidative stress in cancer cells in response to hypoxic stress and outlining that hypoxic cancer cells could maintain intracellular redox homeostasis (Fig. 7g).

Glutathione system and thiorexoxin/glutaredoxins system all need NADPH to regenerate GSH or TrxSH2/GrxSH2 to ensure reduced intracellular microenvironment and GPX function. NADPH levels in hypoxic HepG2 cells are significantly higher than those in hypoxic L02 cells. Pentose phosphate pathway (PPP) is the main source of NADPH. G6PD, the key enzyme of PPP, is inhibited in both hypoxic HepG2 and L02, whereas its activity drops far more rapidly in L02 cells than HepG2 cells. It suggests that NADPH may come from other sources. It is known that NADPH can also be produced from IDH2, a key enzyme involved in TCA cycle^27^. Interestingly, IDH2 expression in hepatoma cells exhibits an unusual increase after hypoxia, suggesting that IDH2 collaborates with the antioxidant system to regulate NADPH production and redox homeostasis in hypoxic cancer cells (Fig. 8e).

### Concluding remarks

Tumor cells not only have higher ROS levels, but also have a higher antioxidant capacity. In hypoxic state, the powerful antioxidant system renders cancer cells rapidly converting hypoxia-induced superoxide anion, effectively keeping ROS below lethal values. On the contrary, such moderate level of ROS can be exploited by cancer cells to induce an appropriate degree of autophagy, which eliminates the damaged mitochondria on one hand, and offers nutrients to promote mitochondria fusion on another hand, so that the mitochondria function can be remained even when oxygen deprivation and glycolysis level increases. Therefore tumor cells could exploit the advantages of both glycolysis and oxidative phosphorylation to promote their hypoxia survivability. When tumors generate blood vessels and experience hypoxia/re-oxygenation cycling, cancer cells can flexibly switch from glycolysis to oxidative phosphorylation to meet the requirements for rapid tumor growth. This study could provide new clues to cancer treatment.

## Materials and Methods

### Cell culture and oxygen concentration

L02, HepG2, SMMC-7721, Huh7 hepatocarcinoma cells, HuvEc, HDF and MCF-10A, triple-negative MDA-MB-231 breast cancer cells were grown in DMEM (Hyclone) supplemented with 2 mM L-glutamine and 10% FBS (Gibico) in a humidified incubator at 37 °C and 5% CO_2_. MCF-10A cells were also supplemented with 0.05 mg/ml gentamicin (Invitrogen). Hypoxia exposures were done in a tri-gas tissue culture incubator (Binder). The oxygen concentration was assayed as previous described^45^. All cells were purchased from the Type Culture Collection of the Chinese Academy of Sciences, Shanghai, China. This study was approved by the Research Ethics Committees of Fudan University and the methods were carried out in accordance with the approved guidelines (You can use the following link: http://www.nature.com/srep/policies/index.html#experimental-subjects to see the details of our guidelines).

### RT-PCR

Cell’s total RNA was isolated by TRIZOL reagent (Invitrogen), according to manufacturer’s instructions. Reverse-transcription reaction was carried out using ABI PRISM 7900 (Applied Biosystems). Primers were designed (Supplementary Table. S1) and purchased from Generay Biotechnology.

### Immunoblotting

Total cell extracts or nuclear extracts were separated by SDS-PAGE and transferred to PVDF membranes. The following antibodies were used for immunoblot analysis: anti-HK2, anti-PFKM, anti-LDHa1, anti-PDK2, anti-PDHa1, anti-ATP5B, anti-IDH2, anti-HIF1α, anti-MnSOD, anti-Cu/ZnSOD, anti-Catalase, anti-GR, anti-GRX1, anti-GPX1, anti-PRX3 and anti-TRX2 antibodies and secondary antibody were purchased from Proteintech. Anti-MnSOD (acetyl K68) antibody was purchased from Abcam. Anti-β-actin, anti-ComplexV, anti-Lc3B, anti-MFN1, and anti-Fis1 antibodies were from Cell Signaling Technology. Protein expression is visualized on Tanon-5200 Chemi-luminescent Imaging System (Tanon Science Technology).

### Immunofluorescence

Cells were washed with PBS twice and fixed by 4% paraformaldehyde. Anti-SOD2 antibody or Anti-SOD2 (acetyl K68) antibody were added into the cell culture dish used for cofocus at 4 °C overnight. After washing with PBS, anti-rabbit antibody containing FITC was hatched for 4 h. The immunofluorescence was analyzed using LEICA SP5 cofocus microsystem.

### Flow cytometry

For measurement of intracellular ROS, mitochondrial O_2·_^−^ production, hydroxyl radical or mitochondrial membrane position (MMP), cells were harvested and incubated with 10 μM DCFH-DA (2′-, 7′-dichlorofluorescein diacetate, Sigma) for 30 min, 5 μM MitoSOX Red mitochondrial superoxide indicator (Invitrogen) for 10 min, 5 μM HPF (3′-(p-hydroxyphenyl) fluorescein, Invitrogen) for 60 min or 5 ug/ml JC-1 probe (Beyotime) for 20 min at 37 °C. Cell suspension solution was centrifuged with 3000 rpm for 5 min, and washed twice with PBS. The fluorescence intensity was analyzed by FC 500 MCL system (Beckman coulter) immediately at excitation/emission wavelength of 488 nm/525 nm (for ROS), 510/580 nm (for mitochondrial O_2·_^−^) or 490/515 nm (for ·OH). The MMP was determined using the ratio of fluorescence value at 595 nm and 525 nm.

### Measurement of ATP and ADP

Intracellular ATP and ADP were measured using high performance liquid chromatography (HPLC) as previously described^46^.

### Enzyme activities

Aconitase, MnSOD and G6PD activity were measured as previously described^47^,^48^. Catalase activity assay was carried out using a chemiluminometric detector (Lumat LB9507, Berthold). · OH was generated by adding 2.68 mM H_2_O_2_ into the reaction system consisting of 0.15 mM CoCl_2_ and 54 μM luminol. The drop of luminescence within 2 min was recorded as the relative catalase activity.

### NADP(H) and GSH assay

Cells were scraped into 200 μl cold 40 mM NaOH for NADPH detection and 50 μl 1 M HPO_3_ for GSH detection. After 2–3 freeze/thaw cycles, the suspension was centrifuged with 12,000 rpm for 10 min at 4 °C and then assayed as previously described^49^,^50^.

### Primary isolation, MTT assay, TEM sample preparation and apoptosis assays

Standard methods were used for primary mouse hepatocytes isolation, MTT assay, TEM sample preparation and Annexin V assay with details in Supplementary Materials and Methods.

### Statistical analysis

The results are reported as means ± standard error. Statistical significance was determined using Student’s t-test and ANOVA with alpha = 0.05.

## Additional Information

**How to cite this article**: Li, P. *et al*. Redox homeostasis protects mitochondria through accelerating ROS conversion to enhance hypoxia resistance in cancer cells. *Sci. Rep.* **6**, 22831; doi: 10.1038/srep22831 (2016).

## Supplementary Material

Supplementary Information
