# Supplementary material for: Redox homeostasis protects mitochondria through accelerating ROS conversion to enhance hypoxia resistance in cancer cells
Source: Sci Rep. 2016 Mar 9;6:22831. doi: 10.1038/srep22831 (PMC4783784; doi:10.1038/srep22831)
Supplement: Supplementary Information [file srep22831-s1.pdf]

Supplemental Information

Redox homeostasis protects mitochondria through accelerating ROS conversion to enhance hypoxia resistance in cancer cells

Pengying Li, Dongyang Zhang, Lingxiao Shen, Kelei Dong, Meiling Wu, Zhouluo Ou, Dongyun Shi

Supplemental Methods

The primary isolation and culture of mouse hepatocytes

Primary mouse hepatocytes were isolated following perfusion of whole liver first with perfusion buffer (Hank's Balanced Saline, HBSS) and then with collagenase solution (HBSS with 1% BSA and 0.05% collagenase) for 2 min. Dispersed cells were resuspended and seeded onto collagen-coated plates in DMEM supplemented with 10% fetal bovine serum in the presence of 1% insulin-transferrin-selenium, 105 unit/L Penicillin and Streptomycin. Four hours after plating, the medium was changed to a fresh DMEM media as described above. All the experiments were approved by the Research Ethics Committees of Fudan University and the methods were carried out in accordance with the approved guidelines (You can use the following link: http://www.nature.com/srep/policies/index.html#experimental-subjects to see the details of our guidelines).

MTT assay

1×10^4 cells were seeded in triplicate in a 96-well plate in a final volume of 100μl and incubated for 4h. Cells treated with DMSO alone were used as controls. Intervention buffer (100μl) was then added and cultured the cells for the indicated times. At the end of the treatment, 10μl MTT (5mg/ml) was added to each well and incubated for an additional 4h at 37℃. DMSO (100ul)/ well were added after dropping the old medium with MTT. The absorbance was measured at 570nm using a microplatereader (Biotek Synergy 4).

TEM sample preparation

The cells were scraped into 500μl 2.5% glutaraldehyde and then centrifuged at 3000 rpm/min at 4℃ for 15min and remove the supernatant. The subsequent manipulations were dehydration by acetone, embedding by embedding medium, sectioning and dyeing. The transmission electron microscope was used for observing the morphology and ultrastructure of mitochondria.

Annexin V/propidiumiodide (PI) staining

Cells were cultured in normoxia or hypoxia incubator for 24 or 48h in 6-well plates and harvested in PBS. Apoptotic cells were identified by double staining with FITC-conjugated annexin V and PI according to the manufacturer's instructions (Beyotime, Shanghai, China). Data were obtained and analyzed using a FC 500 MCL system (Beckman coulter).

Table S1. Primer sequences used for quantitative Real Time PCR.

β-actin	Forward 5'- ATGTGGCCGAGGACTTTGAT -3'
	Reverse 5'- AGTGGGGTGGCTTTTAGGATG -3'
HPRT	Forward 5'- CCTGGCGTCGTGATTAGTGA -3'
	Reverse 5'- AGCAAGACGTTCAGTCCTGT -3'
PKM2	Forward 5'- CGATCAGTGGAGACGTTGAAGGA -3'
	Reverse 5'- GGTAGAGGATGGGGTCAGAAGCA -3'
IDH2	Forward 5'- CGACTTTCAAAATGGTCTTCACC -3'
	Reverse 5'- ATTTCTTCTGGATGGCACACTGGA -3'
IDH3A	Forward 5'- CCAGAAGGGCTGAGTGTATTGTAA -3'
	Reverse 5'- TTGGTGCTTTGGGATAGGAGAAG -3'
SDHA	Forward 5'- CGGAAGGATGTCGTGGAGAG -3'
	Reverse 5'- CATGGACCGAGACACCACAT -3'
Trx2	Forward 5'- CCTGGGTCCCAGAGTGAAAT-3'
	Reverse 5'- AGGAGGCACCTTGAGACTTC -3'
Gpx	Forward 5'- GCAACCAGTTTGGGCATCAG -3'
	Reverse 5'- CACCGCTTCACCTCGCACTTC -3'
MnSOD	Forward 5'- CCGACCTGCCCTACGACTA -3'
	Reverse 5'- GATGGCTTCCAGCAACTCCC -3'
ATP5B	Forward 5'- TGCTGAGCTGGGCATCTATC -3'
	Reverse 5'- TGCTGAGCTGGGCATCTATC -3'
HIF1α	Forward 5'- TGGTGGTTACTCAGCACTTTTAGA -3'
	Reverse 5'- AAATCTCCGTCCCTCAACCTC -3'
Nrf2	Forward 5'- CTCCTACACCAACGCCTTTC -3'
	Reverse 5'- TCGCCCGCGAGATAAAGAG -3'

All values were the mean±SD of at least three experiments. (*p<0.05, **p<0.01, ***p<0.005 *versus* corresponding L02 groups)

Table S2. ATP/ADP ratio in different cell lines under normoxia or hypoxia.

	21% O_2	0.2% O_2
Primary hepatocell	9.69±1.16	1.73±0.12**
L02	12.37±0.78	3.30±0.19
HepG2	12.61±0.49	5.66±0.33**
SMMC-7721	10.11±0.27*	7.49±1.06*
Huh7	9.14±0.05**	9.62±0.66***
MDA-MB-231	8.20±0.59***	10.54±0.13***

All values were the mean±SD of at least three experiments. (*p<0.05, **p<0.01, ***p<0.005 versus corresponding L02 groups)

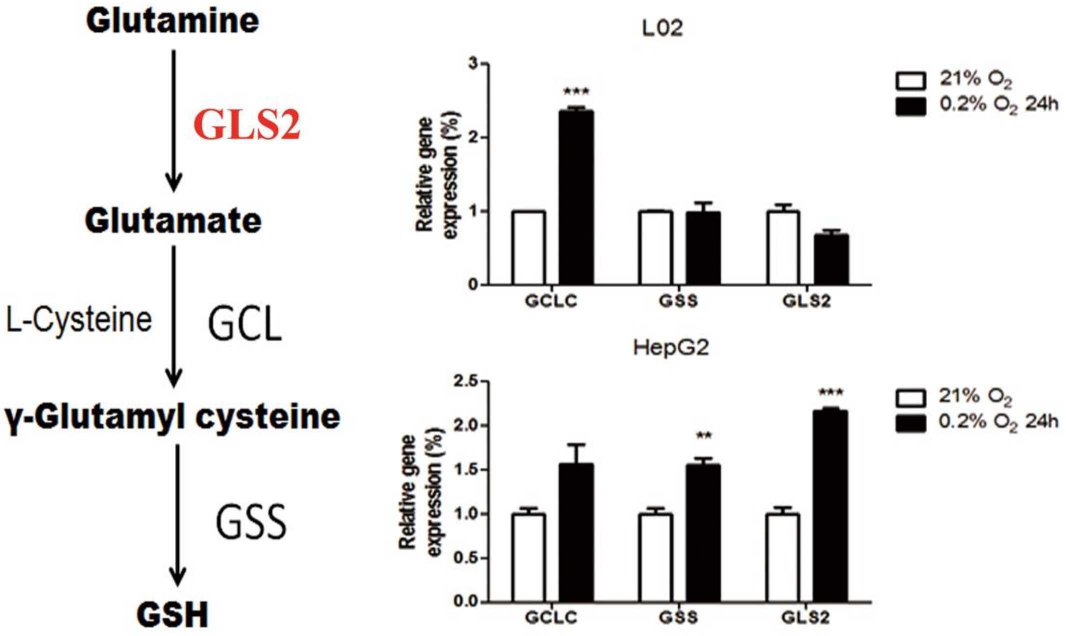

Figure S1. RT-QPCR analysis of GCLC, GSS and GLS2 expression. L02 and HepG2 cells were cultured in 20% or 0.2% O_2 incubator for 24h. mRNA levels were normalized to HPRT mRNA levels. Results are expressed as fold changes from control. Results are shown as mean±SD, n≥3, $^{**}p<0.01$, $^{***}p<0.005$ *versus* normoal.

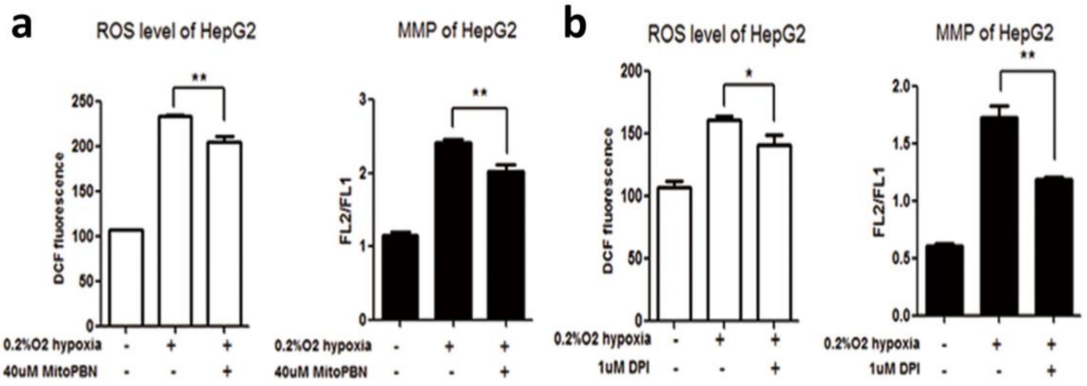

Figure S2. ROS (left part) and MMP (right part) were inhibited by (**A**) MitoPBN or (**B**) DPI in hypoxic HepG2 (0.2% O_2, 24h). Results are shown as mean±SD, n≥3, *p <

0.05, **p<0.01, ***p<0.005.

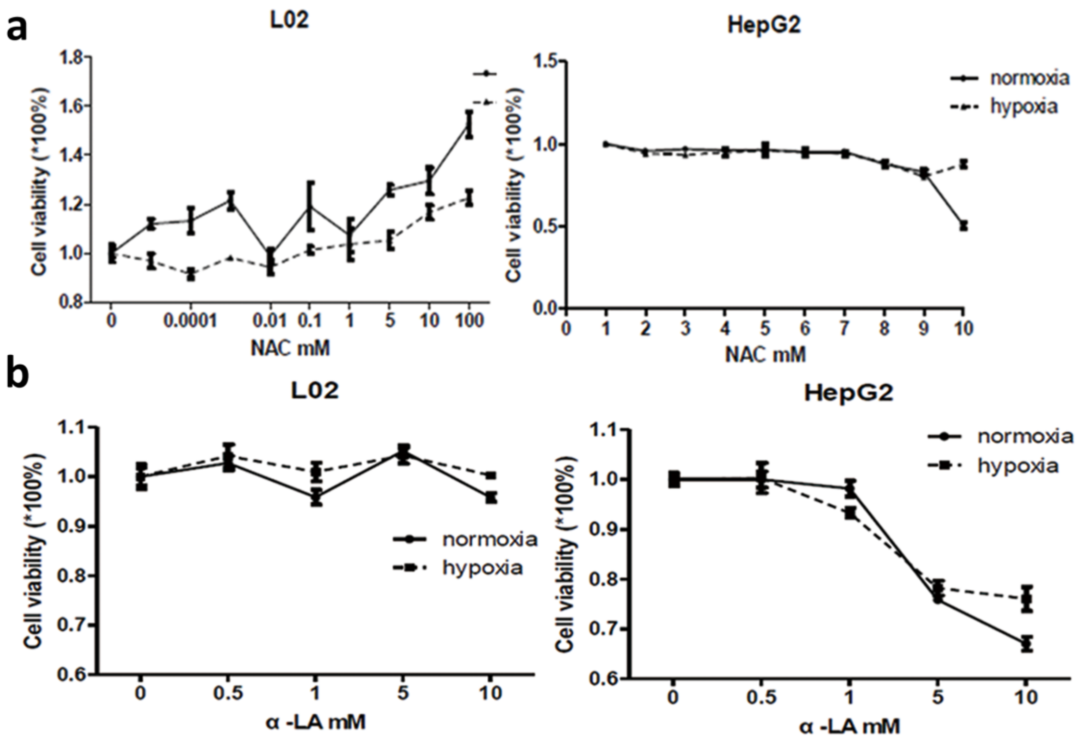

Figure S3. L02 and HepG2 cells were treated with (**A**) NAC or (**B**) α-LA for 2h and then cultured in 0.2% O_2 incubator for 24h. Cell viability was assessed using MTT assay. Results are shown as mean±SD, n≥3.
